# Removal Efficiency of Insoluble β-Cyclodextrin Polymer from Water–Soluble Carcinogenic Direct Azo Dyes

**DOI:** 10.3390/polym15030732

**Published:** 2023-01-31

**Authors:** Lamia Moulahcene, Mohamed Skiba, Nicolas Milon, Hammache Fadila, Frédéric Bounoure, Malika Lahiani-Skiba

**Affiliations:** 1 Galenic Pharmaceutical Laboratory, Normandie University, UNIROUEN, NorDICInserm U1239, UFR Medicine and Pharmacy, Rouen University, 22 Bd Gambetta, F-76183 Rouen, France; 2 Laboratory of Membrane Processes and of Separation and Recovery Techniques, Faculty of Technology, Abderrahmane-Mira University, Route de Targua Ouzemmour, Bejaia DZ-06000, Algeria; 3 Departement of Process Engeneering Technology, Institute of Technology, Akli Mohand Oulhadj University of Bouira, Bouira 10000, Algeria; 4 Departement of Process Engeneering Technology, Faculty of Applied Science, Akli Mohand Oulhadj University of Bouira, Bouira 10000, Algeria

**Keywords:** cyclodextrin polymer, methyl violet, eriochromblack T, helianthin, wastewater treatment, textile dyes, adsorption

## Abstract

A batch system was applied to study the adsorption of three dyes (methyl violet, eriochrom black T and helianthin) from aqueous solution onto β-cyclodextrin polymer, synthesized by using citric acid as a cross linking agent. This polymer lets to adsorb only methyl violet for this effect, several operator variables was checked only with this kind of dye, the removal efficiently increases with increase in adsorbent amount; elevation of temperature lets also to improve the dye adsorption; ionic strength has not effect on dye adsorption process, for the pH we have remarked a slight decrease in removal efficiently with increasing of pH values. Equilibrium study was investigated by applying three models (Langumir, Frendlich and Temkin), results show that Langumir isotherm is the appropriate model. FTIR spectra show the complex inclusion formation which dominates the adsorption mechanism, confirmed by the absence of characteristic peaks of methyl violet in ß-cyclodextrin after adsorption.

## 1. Introduction

Dyes are an important class of organic molecules used by many industries, like plastics, paper, textile, and cosmetics, in order to color their products. These molecules are often found in trace quantities in industrial wastewater, their presence in water even at small concentration is highly visible and undesirable. Many dyes are difficult to degrade; they persist in the environment to cause problems to fauna, flora and public health.

Currently, the textile dyeing industry is one of the most polluting of all industries in the world. It is estimated that the textile industry uses more than five trillion liters of water per year, with effluents from textile processing and dyeing responsible for 20% of the pollution of water. The direct toxic effect of dyes and their metabolites on various living species, including humans, has been established. Due to their xenobiotic effects and persistence, azo dyes like methyl violet; eriochromblack T; helianthin have long-lasting disruptive effects on ecosystems. While these ecosystems have their potential natural remediation pathways, the process does not consistently produce non-toxic or less toxic degradation products. Indeed, some environmental processes promote the transformation of hazardous dyes into toxic metabolites, such as aromatic amines, diazonium and nitrenium ions, and hydrolyzed products. In another way, the microflora of the skin or the intestinal ecosystem of mammals transforms some non-toxic dyes into toxic carcinogens. These end compounds also confer toxicity at different levels of biological compartments via different pathways.

Dyes removal from water has been a challenge since long time; several methods have been reported and attempted for the removal of these pollutants like as membranes [1], photodegradation [2] and adsorption [3], among decolorization technique adsorption gives best results and it is an economically feasible process for purification. Investigation of adsorption equilibrium is very important because their data are the basis for the selection of suitable adsorbent, design of separation process.

Several kinds of absorbent exist; recently a large variety of non-conventional adsorbent is defined such as clay materials [4], agricultural waste and biomass [5,6]. In this way biopolymers and natural molecules like cellulose [7] chitosane [8], starch and cyclodextrin [9,10] are lately very used to remove toxic molecules from water, some adsorbents like activated carbon is difficult to be regenerate, for this the use of cyclodextrin polymers like adsorbent is very interesting because they present many regeneration cycle [9].

β-cyclodextrin (β-CD) materials, as adsorbents for the removal of micropollutant organics. β-CD is a natural, nontoxic, and inexpensive cyclic oligosaccharide produced from starch by enzymatic conversion, 29, 30 and has shown great potential in adsorptive removal of organic micropollutants from wastewater. Traditional β-CD-based polymers are treated for chemical crosslinking with toxic epichlorohydrin and other small linkers to improve their chemical and physical properties. The β-CD-based polymers with short linkers have little nanoframework in addition to the inherent CD cavities, limiting the access of adsorbate molecules and thus the adsorption capacity and efficiency. Recently, cross-linked β-CD-based polymers by citric acid have been reported with a large surface area and mesoporous structure capable of rapidly removing a variety of organic micropollutants. However, these polymers of cyclodextrin were fairly expensive to manufacture, which reduced their potential in practical applications.

Cyclodextrins are cyclic oligomers composed of 6, 7 or 8 glucose units, respectively, termed α, β and γ cyclodextrin. Cyclodextrins are known for their ability to include various molecules in their hydrophobic cavity, in particular allowing solubilization in water and biological environments of molecular structures little or not soluble in these mediums and if required, to improve their stability and bioavailability. The structure of the cyclodextrins may be shown as a conical trunk with a hydrophobic cavity. The outside of the cyclodextrin molecule is generally hydrophilic, these are pseudo-amphiphilic molecules. Such pseudo-amphiphilic structure may allow the formation of inclusion complexes. The inclusion complex may have physico-chemical properties, which are independent from the guest molecule and thus improve the apparent water solubility of this molecule.

Native cyclodextrins (CD), because of their low solubility in water: 127 g/L for α-CD, 18.8 g/L for β-CD and 236 g/L for γ-CD, can have be limited in their use, in particular in the case of β-cyclodextrin. In order to solve this, highly soluble modified cyclodextrins and amorphous structures may be used. The presence of hydroxyl groups on the native cyclodextrins make it possible to develop cyclodextrin derivatives having an improved solubility. Indeed, native cyclodextrins have three types of alcohol groups: a primary alcohol group by molecular structure of glucose (position 6) and two alcohol groups by molecular structure of glucose (position 2 and 3), which represents 21 alcohol groups for β-CD capable of reacting. Among these derivatives, partially or completely methylated cyclodextrins have a significantly improved water solubility compared to native cyclodextrins. In addition, methylated cyclodextrins retain the properties of native cyclodextrins and in some cases can improve them with the extension of the cavity using substituted hydrophobic methyl groups. According to the size of the host molecules, their inclusion in the cavity of cyclodextrins is limited. For example, larger molecules, especially macromolecules, proteins and peptides are generally not suitable for inclusion in cyclodextrins. In addition, the molar ratio of cyclodextrin to host molecule is generally 1/1 or higher.

In contrast, cyclodextrin polymers have several advantages. For example, they have higher molecular weights than cyclodextrins, the macromolecular structure of cyclodextrin polymers means that they can be considered to be biomaterials and the stability constants of the polymer-substrate complexes are often larger than those of cyclodextrin-drug complexes. As a result, hydrophobic, hydrophilic compounds and supramolecules are more readily complexed and less readily released by cyclodextrin polymers than by native cyclodextrins.

Insoluble cyclodextrin polymers is a non-conventional adsorbent, it can be obtained using cyclodextrin (CD) as complex molecule and bi or polyfunctional substance as cross-linking agent [11], this adsorbent is very used in dyes removal from wastewater with best results [10,11,12,13,14].

In this paper we propose the use of cyclodextrin polymer (synthesized by combination β-cyclodextrin and citric acid as a cross-linking agent) as adsorbents of methyl violet, Helianthin and Eriochrom blackT, for water treatment applications. The influence of several parameters (kinetic contact time, adsorbent amount, initial pH, ionic strengh, temperature and dye concentration) has been investigated, the equilibrium data have been analyzed using three models (Langumir, Frendlich and Temkin), Langumir is the appropriate model for description of the methyl violet adsorption on ß-cyclodextrin polymer.

## 2. Materials and Methods

### 2.1. Reagent

**Figure 1:**
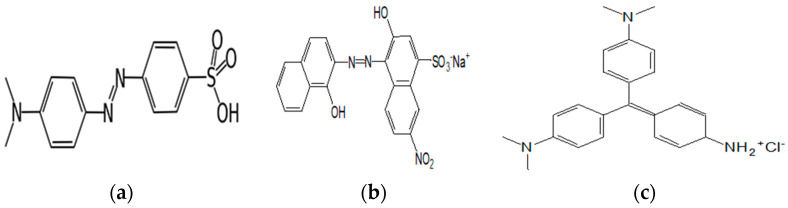
Chemical structure of (**a**) Hélianthin, (**b**) Eriochrom black T, (**c**) methyl violet.

The methyl violet, Helianthin and Eriochrom blackT were purchased from Sigma (Figure 1), insoluble β-cyclodextrin polymer (P-β-CD) was acquired from start-up In-Cyclo, University of Rouen-France. chloridric acid, sodium chloride and sodium hydroxide were obtained, respectively, from Chemi-nova, Labosi and Merck KGaA.

### 2.2. Apparatus

IR spectra were obtained with Perkin-Elmer 4000 FTIR spectrometer, the scanning was from 4000 cm^−1^ to 400 cm^−1^. UV-Visible analysis was carried out with JASCO, V-R30, JAPAN spectrophotometer, a glass tank is used, and methyl violet solutions are prepared in deionized water, the measurements are carried out at 550 nm, 530 nm and 461 nm for, respectively, methyl violet, eriochrom Black T and Helianthin. The morphology of the polymer was determined using scanning electron microscopy Cambridge Steroscan 360 by using the LFD mode.

### 2.3. Preparation of Polymer

**Figure 2:**
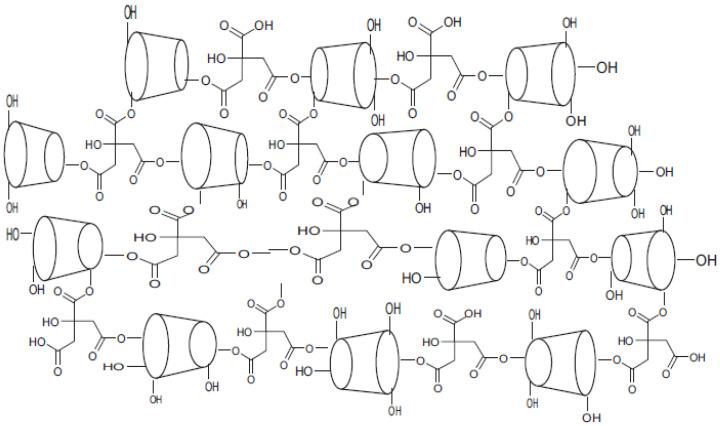
Cross-linked structure of cyclodextrin polymer.

ß-cyclodextrin polymer (Figure 2) is synthesized by direct melt copoly-condensation, according to the method reported by Skiba [11,12,13,14,15] briefly, a mixture of known amount of β-cyclodextrins, citric acid and sodium phosphate dibasic was transferred into a reactor which was maintained at temperature ranging between 140 and 150 °C for fixed time. The obtained solid form was dissolved in water and dialyzed using polyether sulfate membrane filter with molecular weight cut off of 10,000 Da. After the dialysis, the resulted solution was spray dried using BUCHI Mini Sprayer Dryer B-290, two fractions were obtained soluble and insoluble polymer. The insoluble polymer which is used as an adsorbent in this work, was washed with methanol and dried at 60 °C.

### 2.4. Adsorption Studies

Adsorption experiments were carried out in a batch system, 100 mL of dye solution was introduced in a glass bottle, a quantity of cyclodextrin polymer was then added and stirred, at an interval of time, 3 mL of the solution was taken; the dye solution was then separated from the adsorbent by centrifugation at 2000 rpm for 5 min and analyzed by UV-Visible spectrophotometer.

Stock solution of dyes (2.5 10^−5^ mol.L^−1^) was prepared in deionized water. The experimental solutions with desired dyes concentrations were obtained by successive dilution of this stock solution with deionized water. Calibrations curves of dyes were prepared by measuring absorbance of samples with predetermined concentrations at 550 nm, 530 nm and 461 nm for, respectively, methyl violet, eriochrom black T and helianthin (corresponding to a maximum absorbency of dyes) using UV/VIS spectrophotometer (JASCO, V-R30, Japan).

### 2.5. Adsorption Isotherms

The most common relation between the adsorbate concentration and the adsorbed amount is adsorption isotherm, which was measured at room temperature. 20 mg of the β-cyclodextrin polymer was mixed with 50 mL methyl violet solution with different concentrations (1.10^−4^, 0, 9.10^−4^, 0, 8.10^−4^, 0, 7.10^−4^, 0, 5.10^−4^ and 0, 4.10^−4^ mol.L^−1^), the mixture was then stirred for 24 h according to the kinetics experiments. In this work three mathematical models were used for modeling of experimental data (Langumir, Frendlich and Temkin).

## 3. Results and Discussion

### 3.1. Cyclodextrin Polymer Characterization

**Figure 3:**
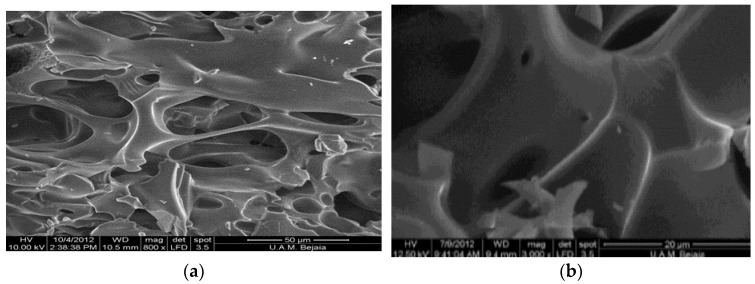
Feature (SEM) of ß-cyclodextrin polymer at (**a**) 800× magnification and (**b**) 3000× magnification.

Feature at 800× magnification corresponding to SEM analysis of β-cyclodextrin polymer is represented in Figure 3, it has a porous structure with homogenous and smooth cavities, and the same structure was reported with the copolymer cyclodextrin-polyamidoamine synthesized by Li et al. [16].

Some proprieties of β-cyclodextrin polymer determined by Moulahcene et al. [9] are presented in the following Table 1:

As it is reported by L. Moulahcene et al. [9] β-cyclodextrin polymer presents high swelling capacity due to its porous morphology confirmed by the SEM feature (Figure 3), this polymer presents also a small surface area, this effect indicates that the adsorption mechanism is different from those of other conventional adsorbents, the same results were found in literature [16], the number of total acidic groups is relatively high, which lets the β-cyclodextrin polymer to have an important physicals interactions in the polymer network to adsorb dyes.

### 3.2. Adsorption of Dyes onto β-Cyclodextrin Polymer

**Figure 4:**
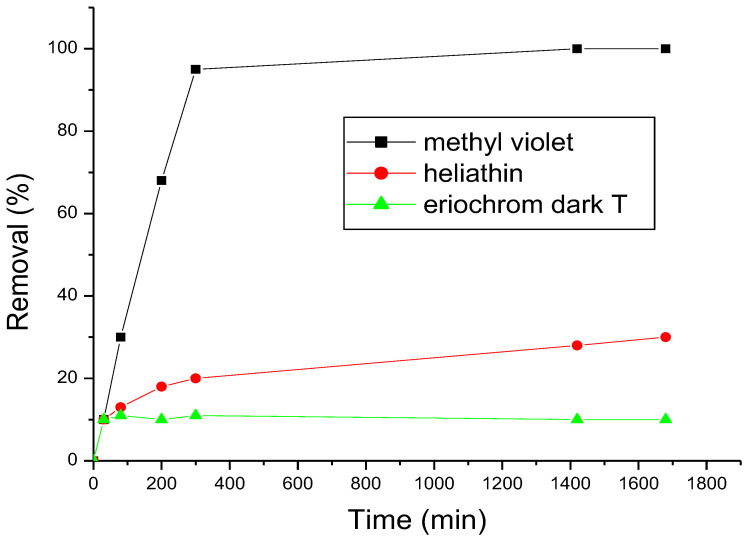
Adsorption of the three dyes on ß-cyclodextrin polymer, dyes concentrations 2.5 10^−5^ mol.L^−1^, ß-cyclodextrin polymer amount 20 mg, T = 30 °C.

Adsorption of the three types of dyes is presented in the Figure 4, we note that helianthin and eriochrom black T have not affinity to the β-cyclodextrin polymer, which gives low efficiently equals, respectively, to 10% and 30% but with methyl violet we attain an efficiently of 100%. This effect may be explained by the big size of the two molecules, compared to the β-cyclodextrins cavity size which can’t form inclusion complexes, this effect may be also due to the hydrophilic character of the two molecules, which is unfavored to inclusion complexes formation with β-cyclodextrins in the polymer, in case of methyl violet his molecular size lets to form inclusion complexes with β-cyclodextrins in the polymer and it is less hydrophilic. For this reason we have chosen methyl violet for further investigation.

### 3.3. Comparison between Native β-Cyclodextrine and β-Cyclodextrin Polymer

**Figure 5:**
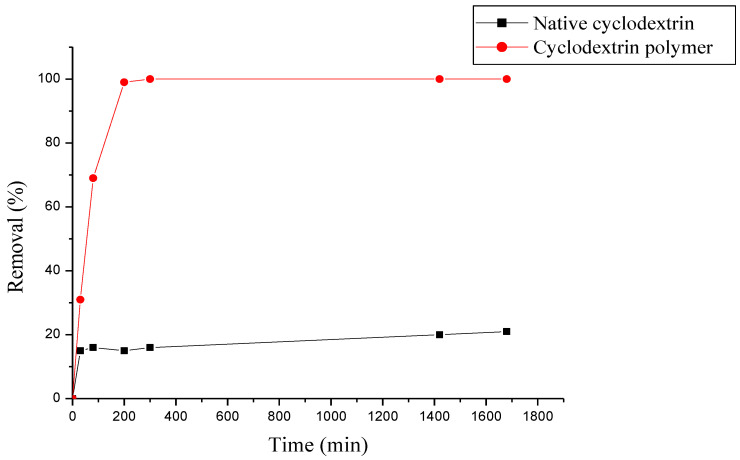
Comparison study of methyl purple removal with native β-cyclodextrin and β-cyclodextrin polymer, T = 30 °C.

A comparison study of methyl violet removal by using native β-cyclodextrine and β-cyclodextrin polymer was done; results are mentioned in Figure 5, we remark an important difference between dye removal by native β-cyclodextrin and β-cyclodextrin polymer, this last gives the best efficiency with nearly 100%, but in case of native cyclodextrin it gives only 20%. Methyl violet is able to form inclusion complexes with β-cyclodextrin units in aqueous solution but these complexes are very soluble and unstable, removal of methyl violet with native β-cyclodextrin is not interesting.

### 3.4. Effect of Operators Variables on Methyl Violet Adsorption

#### 3.4.1. Effect of pH and Ionic Strength

**Figure 6:**
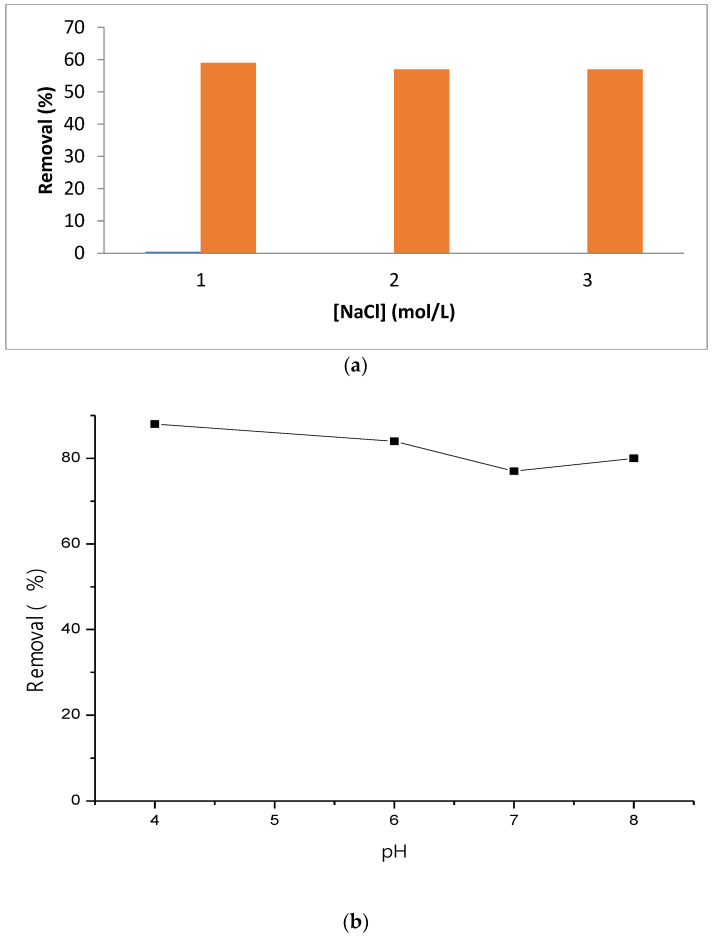
(**a**) ionic strength, (**b**) Effect of pH on methyl violet removal by ß-cyclodextrin polymer, T = 30 °C.

Effect of pH on methyl violet adsorption is shown in Figure 6b, methyl violet is not undissociated under pH condition, we remark a slightly decrease in methyl violet adsorption with increasing of pH, this effect may be attributed to the substitution of Cl by -OH groups which decreases the capacity of methyl violet to form inclusion complexes with cyclodextrins, with other adsorbent reported in literature like as cellulose, the pH play a crucial role in methyl violet adsorption due to the positive charge in methyl violet [7]. The ionic strength is studied by using NaCl with a concentration between 0 and 3 M. Results are presented in Figure 6a, ionic strength also does not have effect on methyl violet adsorption.

#### 3.4.2. Effect of Adsorbent Amount

**Figure 7:**
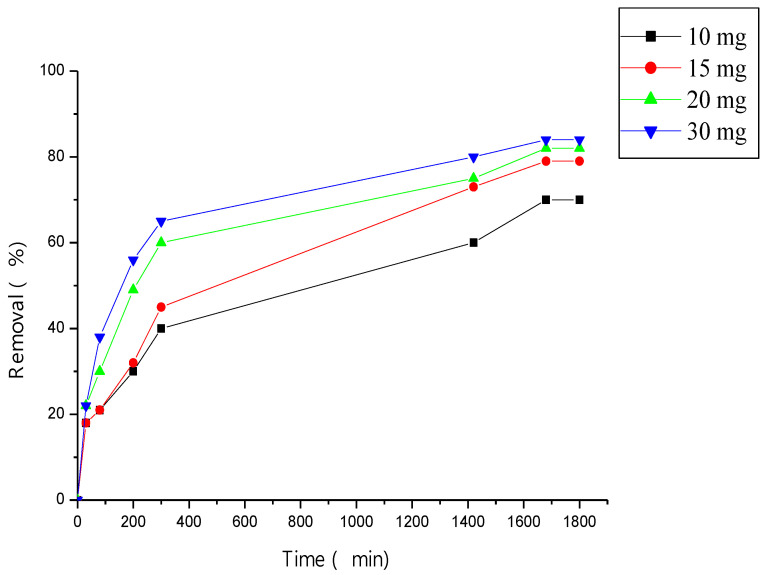
Effect of ß-cyclodextrin polymer amount on methyl violet removal, pH = 4, T = 30 °C.

The effect of adsorbent amount on methyl violet adsorption is represented in Figure 7, the efficiently increases with increase in adsorbent amount and the equilibrium is attained at 1700 min.

#### 3.4.3. Effect of Temperature

**Figure 8:**
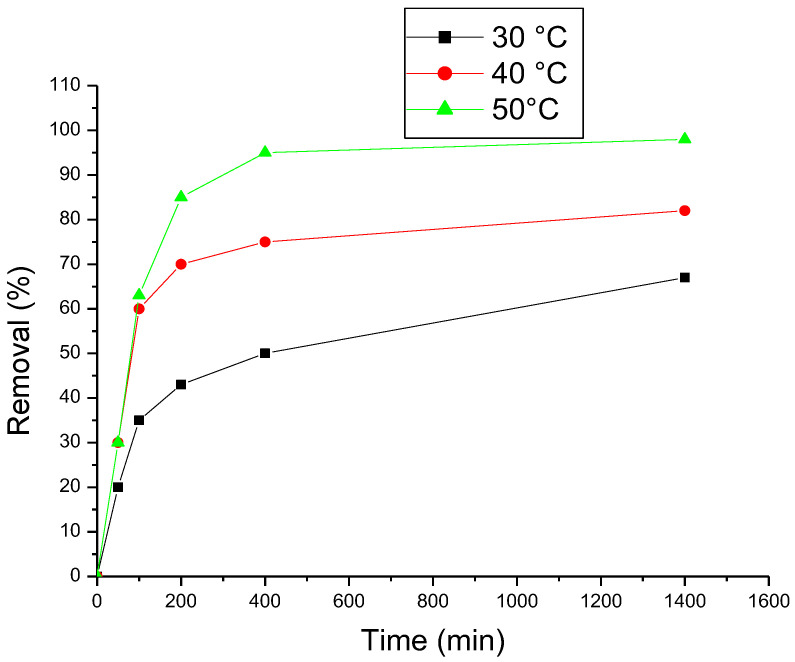
Effect of temperature (T) on methyl violet removal.

Temperature has an important effect on methyl violet adsorption onto ß-cyclodextrin polymer, results are shown in Figure 8, adsorption kinetic is improved with increasing of temperature, this effect may be attributed to increasing of the intraparticle diffusion rate or to enlargement of pore size [9,10,11,12,13,14,15,16,17].

### 3.5. Adsorption Isotherms

Figure 8 shows adsorption isotherm for ß-cyclodextrin polymer using methyl violet, the equilibrium adsorption capacity (*q_e_*), increased with increase in dye concentration. The shape of the isotherm indicated an L-behavior according to Giles et al. [18] classification, which confirms a high affinity between the β-cyclodextrin polymer and the dye molecule [3].

The experimental adsorption equilibrium data of methyl violet onto β-cyclodextrin polymer is studied by using three models (Langumir, Frendlich and Temkin). The different equations of the three isotherms are mentioned below:

The Langmuir isotherm used for homogeneous surfaces and assumes the formation of a monolayer [19] is given by:

(1) $$q_{e}=q_{\max}\times \frac{K_{L}}{1+C_{e}K_{L}}$$

where *C_e_* is the equilibrium dye concentration in the bulk (mg L^−1^), *q_e_* is the amount of adsorbate per unit mass of adsorbent at equilibrium (mg g^−1^), *q_max_* is the monolayer adsorption capacity of the adsorbent (mg g^−1^) and *K_L_* the Langumir isotherm constant (L mg^−1^).

The linear form of the Langumir isotherm is given by the Equation (2):

(2) $$\frac{C_{e}}{q_{e}}=\frac{1}{q_{max}}C_{e}+\frac{1}{q_{max}K_{L}}$$

The second model is the Frendlich one which is applied to heterogeneous surface and assumes that there is not formation of a monolayer and it is given as:

(3) $$q_{e}=K_{F}\times {C_{e}}^{1/n}$$

where *n* and *K_F_* (L^1/*n*^ mg^−1/*n*^ g^−1^) are constants.

The linear form of the Frendlich isotherm is:

(4) $$\ln q_{e}=\frac{1}{n}\ln C_{e}+\ln K_{F}$$

Temkin isotherms consider the effects of some indirect adsorbate/adsorbate interaction on adsorption isotherms. The Temkin isotherm can be expressed in its linear from as [7]:

$$q_{e}=A\cdot \ln{BC}_{e}$$

where *B* is the equilibrium binding constant corresponding to the maximum binding energy (l mg^−1^) and *A* is the Temkin constant related to the adsorption heat.

**Figure 9:**
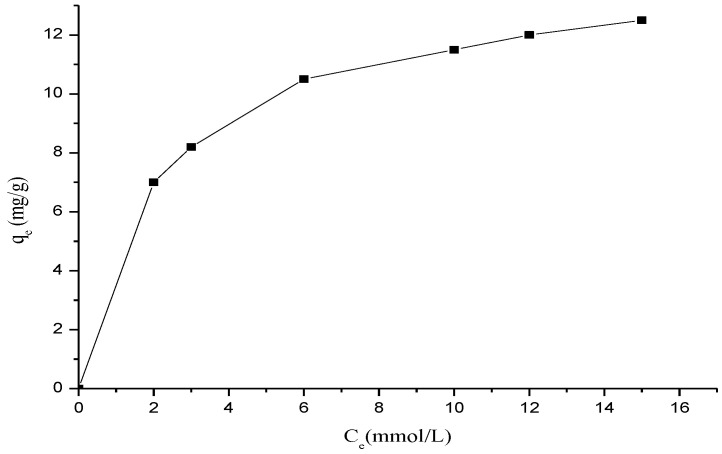
Adsorption isotherm of methyl violet onto cyclodextrin polymer at room temperature, T = 30 °C.

The results are shown in Figure 9, the system obeys Langumir isotherm because the plot of *C_e_*/*q_e_* versus Ce is linear with a good correlation coefficient (r^2^ = 0.9997).

**Figure 10:**
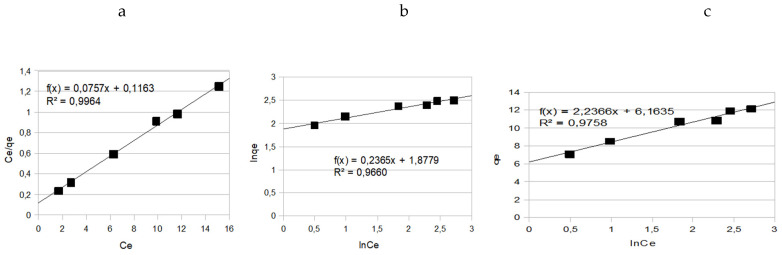
Linear plot of (**a**) Frendlich isotherm, (**b**) Langmuir isotherm and (**c**) Temkin isotherm for methyl violet onto β-cyclodextrin polymer.

Correlation coefficient of the different models, the Langumir constant and the maximum capacity for methyl violet adsorption onto β-cyclodextrin polymer are mentioned in Table 2. And Figure 10. Adsorption of methyl violet onto many other adsorbents cited in literature obeys Langumir isotherm [3,4,5,6,7,8,9,10,11,12,13,14,15,16,17,18,19,20].

### 3.6. Physicochemical Characterization

**Figure 11:**
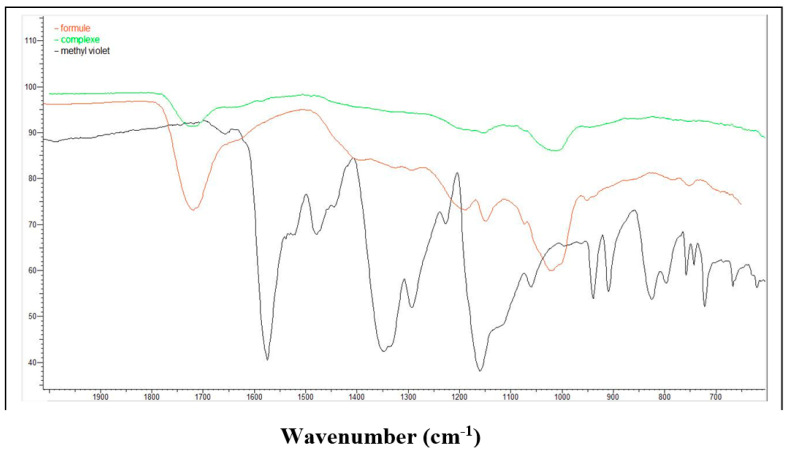
FTIR spectra of methyl violet and β-cyclodexrin polymers after and before adsorption, red (β-cyclodextrin polymer before adsorption), green (β-cyclodextrin polymer after adsorption) and black (methyl violet).

The FTIR spectra (500–4000 cm^−1^) of methyl violet and β-cyclodextrin polymer before and after adsorption are represented in Figure 11. The peak at 1500 cm^−1^ and 1600 cm^−1^ is attributed to the vibration of aromatic C = C bond, and the peak at 1200 cm^−1^ and 1300 cm^−1^ is attributed to the vibration of aromatic amine bond in methyl violet spectra.

The absence of the characteristics peaks of the methyl violet in the β-cyclodextrin polymer before adsorption, confirms the formation of an inclusion complex between β-cyclodextrin and methyl violet, the physical adsorption is neglected, where the extraction mechanism is governed by formation of an inclusion complex between methyl violet and β-cyclodextrin.

## 4. Conclusions

The synthesis of insoluble polymers using β-cyclodextrin as the base material and citric acid as the cross-linking agent was found to be quite viable, yielding a product that could be used as an effective and promising sorbent in the liquid-solid sorption process for the removal of azo dyes from wastewater effluents.

The adsorption of three dyes (methyl violet, eriochrom black T and helianthin) onto β-cyclodextrin polymer has been investigated, methyl violet presents the high adsorption efficiently for this effect, different operators variables has been checked with this kind of dye, removal of methyl violet onto β-cyclodextrin polymer increases with increasing of adsorbent amount and decreases with increasing of adsorbate initial concentration, removal is also increased with temperature, pH has a slightly effect, in contrast ionic strength don’t affects adsorption phenomena. Adsorption equilibrium followed Langumir isotherm, the sharpe of isotherm indicated L-behavior confirming high affinity between the β-cyclodextrin polymer and methyl violet. The adsorption mechanism is dominated by an inclusion complex formation between methyl violet and β-cyclodextrin, results confirmed by the absence of characteristics peaks in FTIR spectra of methyl violet in β-cyclodextrin polymer before adsorption.

The results obtained show that the β-CDs polymer could be used for methyl violet removal as a promising new alternative to other more expensive adsorbents. Its advantages comprise good adsorption properties, ease of compounding and relatively low cost.

This β-CDs polymer could help reduce the environmental impacts caused by the discharge of dye effluents into aquatic systems, while reducing the costs of treating waste for either disposal or reuse in production processes.

## Figures and Tables

**Table 1:** Some proprieties of β-cyclodextrin polymer.

Proprity	Swelling Capacity (%)	Total Acidic Groups (mmol/g)	Surface Area (m^2^/g)
β-cyclodextrin polymer	70.38	9.75	1096

**Table 2:** The constants of isotherm modeling for methyl violet adsorption onto β-cyclodextrin polymer.

Model	Langumir	Frendlich	Temkin
r^2^	0.9997	0.9898	0.9945
*K_L_* (L/mg)	0.65091	-	-
*q_max_* (mg/g)	13.21	-	-

## Data Availability

The data presented in this study are available on request from the corresponding author.
